# An international stratigraphic dataset and geological map of the Ahr River catchment, Germany

**DOI:** 10.1016/j.dib.2023.109677

**Published:** 2023-10-13

**Authors:** Svenja Scholz, Henriette Westermann, Ronja Hegemann, Mara Popp, Tobias Richter, Markus L. Fischer, Christoph Zielhofer

**Affiliations:** a Institute of Geography, Leipzig University, Johannisallee 19a, D-04103 Leipzig, Germany; b Institute for Geoscience, University of Potsdam, Karl-Liebknecht-Str. 24-25, D-14476 Potsdam-Golm, Germany; c LeipzigLab Working Group on Historic Anthropospheres, Leipzig University, Straße des 17. Juni 2, D-04107 Leipzig, Germany

**Keywords:** Geology, Geological Map, International stratigraphic notation, Lithology, Western Germany, Ahr catchment

## Abstract

This dataset provides detailed chronostratigraphic and lithological maps of the entire Ahr River catchment, which is located in Western Germany. The geological information was acquired using a transfer of the German chronostratigraphic terms into the international stratigraphic notation. Information about the geology and lithology was provided by publicly sourced data released by the German federal states North Rhine-Westphalia and Rhineland-Palatinate. The dataset includes information about the international stratigraphy, the corresponding German unit, and the lithology in English and German. The dataset is essentially useful for catchment-scale research, for example with regard to the causes and consequences of the July 2021 flooding of the Ahr River.

Specifications Table

**Table:** 

Subject	Earth and Planetary Sciences – Geology, Stratigraphy
Specific subject area	Standardized international geological map, lithological map, linking German and international stratigraphic notation, catchment-scale approach, GIS analysis
Type of data	Tables, Figures, Maps
How the data were acquired	- Interpreted German geological data were transferred into the international stratigraphy using a sheet that compares German units with the German notation and the international stratigraphy. - QGIS (version 3.24.2.) was used to acquire the new shapefiles and to design the final map. - ArcGis (version 10.3) was used to determine the flow directions, flow accumulations and catchment boundaries.
Data format	raw
Description of data collection	not applicable
Data source location	Raw data location: - https://www.geoportal.rlp.de/spatial-objects/386 (accessed 3 November 2022). - https://www.opengeodata.nrw.de/produkte/geologie/geologie/GK/ISGK100/ISGK100vektor/ (accessed 3 November 2022).
Data accessibility	Repository name: Pangaea https://www.pangaea.de/ Data identification number: https://doi.pangaea.de/10.1594/PANGAEA.959756 Direct URL to data:https://download.pangaea.de/dataset/959756/allfiles.zip See DOI Link https://doi.pangaea.de/10.1594/PANGAEA.959756

## 1 Value of the Data

• Given the background of the catastrophic Ahr River (Germany) flood event in 2021, international geoscientific research projects aim to understand the hydro-sedimentary dynamics, causes, and consequences within the catchment. Profound knowledge about the underlying geology and their lithographic distribution is inevitable to draw conclusions about past, recent, and future hydrological, geomorphological, and environmental processes.
• As research is international, the German stratigraphic notation is insufficient. This dataset provides the first transfer of the German notation into the international stratigraphic system for the Ahr River catchment. The new dataset contains a standardization of the heterogeneous data from two federal states.
• This dataset may serve modelers as input data for drainage, infiltration, and surface flow simulations in combination with e.g., spatial precipitation measurements.
• Geoscientist can use the newly compiled data as a base for sediment provenance analysis in the Ahr River.

## 2 Objective

The Ahr River catchment covers areas of the two federal states North Rhine-Westphalia and Rhineland-Palatinate in Western Germany. Both federal states published information about the geology and lithology but accessibility, structure and usability of the data differs. Furthermore, there is no geological map using the international chronostratigraphic chart, yet, as it is provided in the German notation system only. This dataset targets to combine the information of the two federal states and make them easily accessible and usable. Also, the German stratigraphic notation is transferred into the international system. This dataset comprises a master data sheet, shapefiles, and maps to be used for further projects on the subject by others.

## 3 Data Description

### 3.1 Master data

The master data sheet *Ahr_geology.csv* contains information about the German chronostratigraphic and lithological units that can be found in the Ahr River catchment and their equivalent in the international notation. The master data is provided as shapefile *Ahr_masterdata.shp* as well. For detailed information concerning the master data see Table 1. Information about the German notation and the transfer to the international system stems from the *Stratigraphische Tabelle von Deutschland* (DSK 2016) [4]. Information about the international notation is given by the *International Commission on Geological Sciences* (Cohen et al. 2013, updated) [1].

**Table 1:** Ahr River catchment master data Ahr_geology.csv and Ahr_masterdata.shp description.

Column	Parameter	Description
1	fid	polygon code: random
2	order	sorted in descending stratigraphic order using column 16
3	period_on	period marking the unit's onset according to IUGS 2023
4	period_off	period marking the unit's offset according to IUGS 2023
5	epoch_on	epoch marking the unit's onset according to IUGS 2023
6	epoch_off	epoch marking the unit's offset according to IUGS 2023
7	epoch_gen	combined column for epoch_on and epoch_off
8	stage_on	stage marking the unit's onset according to IUGS 2023
9	stage_off	stage marking the unit's offset according to IUGS 2023
10	symbol	symbol code: Uppercase letters mark epochs and lowercase letters mark stages. Underscores mark timespans comprising multiple epochs / stages, e.g., LDe_MDe comprises the timespan from Lower Devonian (Emsian) until Middle Devonian (Eifelian).
11	dominant	stage that comprises the unit's longest timespan. Epochs were chosen if stages were N.A.
12	lith_eng	lithological information, translated from column 13
13	lith_ger	lithology in German language, provided by GÜK 300 and GK 100
14	lith_gen	categorized lithology
15	unit_ger	original German stratigraphy, provided by GÜK 300 and GK 100
16	DSK_on	numerical age (Ma) marking the unit's onset according to DSK 2016
17	DSK_off	numerical age (Ma) marking the unit's offset according to DSK 2016
18	IUGS_on	numerical age (Ma) of the stage including the unit's onset according to IUGS 2023. Epochs were chosen if stages were N.A.
19	IUGS_off	numerical age (Ma) stage including the unit's offset according to IUGS 2023. Epochs were chosen if stages were N.A.

### 3.2 Shapefiles

The geology and lithology shapefiles are provided as *Ahr_geology.shp* and *Ahr_lithology.shp* and contains the spatial distribution of the different stratigraphic respectively lithological units. The attribute tables are shorter versions of the master table, containing only the columns relevant to the maps (see Tables 2 and 3).

**Table 2:** Ahr_geology.shp: Attribute table description.

Column	Parameter	Description
1	order	sorted in descending stratigraphic order updated after dissolving
2	period_on	period marking the unit's onset according to IUGS 2023
3	period_off	period marking the unit's offset according to IUGS 2023
4	epoch_on	epoch marking the unit's onset according to IUGS 2023
5	epoch_off	epoch marking the unit's offset according to IUGS 2023
6	epoch_gen	combined column for epoch_on and epoch_off

**Table 3:** Ahr_lithology.shp: Attribute table description.

Column	Parameter	Description
1	order	sorted in descending stratigraphic order updated after dissolving
2	period_on	period marking the unit's onset according to IUGS 2023
3	period_off	period marking the unit's offset according to IUGS 2023
4	lith_gen	categorized lithology

## 4 Experimental Design, Materials and Methods

### 4.1 Primary data

The Ahr River is situated in the German federal states North Rhine-Westphalia and Rhineland-Palatinate. Hence, we used geological data provided by the geological services of the individual states. For Rhineland-Palatinate we used the *Geologische Übersichtskarte* (GÜK 300) [2] which is a 1:300 000 vector dataset, whereas the North Rhine-Westphalia dataset is a 1:100 000 vector dataset (GK 100) [3]. Both datasets provide the stratigraphy and the lithology using the regional German notation. However, the GK 100 dataset [3] has a higher spatial resolution and the stratigraphy and lithology are more comprehensive.

The transfer of the German regional stratigraphic terminology to an internationally comprehensible system is done using the DSK 2016 and the *International chronostratigraphic chart* (version 2022/02) [1]. The DSK 2016 sheet compares the regional German and the international stratigraphic terms, providing information about the onset, offset and overlaps of both. We then used the *International chronostratigraphic chart* (version 2023/04) [1] to derive current dates for the international stratigraphic units.

## 5 Creation of the Master Data *Ahr_geology.csv*

First, we created a new shapefile that includes both the information from the GÜK 300 and the GK 100 (using the “Hauptschichten” layer) within the Ahr River catchment. Therefore, we used QGIS (version 3.24.2.). The catchment boundary calculation of the Ahr River was based on a DEM from the Shuttle Radar Topography Mission [5]. The flow directions, flow accumulations, and catchment boundaries were determined using ArcGIS 10.3. For the GK 100 dataset the *Check Validity* tool in QGIS revealed three invalid polygons within the study area. We manually corrected those using the *Toggle Editing Vertex Tool.* Then, we clipped both the GÜK 300 and the geometry-corrected GK 100 dataset with the Ahr River catchment. For the GK 100 only the North Rhine-Westphalia part of the catchment remained, whereas the GÜK 300 covers the whole catchment but the polygons outside Rhineland-Palatinate contain no information. To extract the polygons of interest, several steps were necessary: First, we performed *Difference* on the Ahr catchment shape with the clipped GK 100 as input and *clipped* the GÜK 300 catchment shape with it. Note that for this shapefile several data errors outside the Rhineland-Palatinate part of the catchment have been removed manually using the *Toggle editing Vertex tool*. Then, we *clipped* the GÜK 300 with the GÜK 300 catchment shape.

Next, we dissolved the individual datasets by “symbol” (GK 100) respectively “label” (GÜK 300) to group areas with the same stratigraphy in the attribute table. Note that the symbols for the stratigraphic units differ in the datasets, so we added the GK 100 symbols to the GÜK 300 attribute table manually. Therefore, we identified the individual stratigraphic units in the GÜK 300 legend and compared them to the units in the GK 100 attribute table to find the corresponding symbols. For fid 17 and 60 more than one assignment is possible as the GK 100 dataset provides more detailed information. Hence, we decided not to assign them to a unit in the GK 100 but to keep them separated. Furthermore, the GK 100 polygon concerning bodies of water was deleted (fid 17) as it covered the Rhine and the GÜK 300 polygons for bodies of water were assigned to the surrounding features as they do not cover a water surface.

Then, we merged and dissolved both datasets by “symbol”. Information about the lithology, period, epoch, and stage provided by the GK 100 dataset were matched to the attribute table fields which were derived from the GÜK 300 dataset for all cases but the two described above (fid 17 and 60), where we kept the GÜK 300 information. Next, we corrected some vertices and two polygons of the dissolved shape file by using the *Toggle Editing Vertex Tool.* The result is a complete and unified attribute table of the Ahr River catchment that contains detailed stratigraphic and lithological information using regional German terminology. We exported this attribute table to an excel file and deleted all information but the column “fid” from the attribute table to become the foundation of the master data.

Subsequently, we transferred the regional German stratigraphic system to the international notation. The column “EINHEIT” provided by the GK 100 dataset contained regional German terms for stratigraphic units that correspond with the terms in the DSK 2016 table. Hence, it was possible to derive exact dates for the regional units from the DSK 2016 table and we extended the master data sheet *Ahr_geology.csv* by the columns “DSK_on” and “DSK_off”. We matched these dates with those from IUGS 2023 and found corresponding regional and international terms for the stratigraphic units. On a practical note, we therefore ignored the timespans given by IUGS 2023 for the boundaries between epochs and stages as otherwise too many options for the assigning would have been possible. We added the international terms in the columns “period_on”, “period_off”, “epoch_on”, “epoch_off”, “stage_on” and “stage_off”. Furthermore, we added the dates provided by IUGS 2023 in the columns “IUGS_on” and “IUGS_off” and determined the dominant epochs according to IUGS 2023.

In most cases, the assignment of the international stratigraphy is clear as the regional and the international stratigraphy correspond well. However, there are some cases where we had to decide for an option:

For fid 3 only the Holocene as epoch was given by GK 100, but we decided to add the stage Meghalayan as the given lithology claims this unit to be anthropogenic. Furthermore, we decided to assign the beginning of the units with the fid 10, 13, 38 and 41 to the Pleistocene instead of the Pliocene. We decided to ignore the overlap between the German “Pleistozän” and Pliocene as otherwise units stemming from the Pleistocene would have been assigned to the Pliocene. But due to the major change coming with the transition from Pliocene into Pleistocene only the assignment to the Pleistocene is appropriate. For fid 17 and 36 we assumed that the name “Heisbach”-unit in the information given by GK 100 refers to the German unit “Heisdorf” as no “Heisbach”-unit exists. Fid 37 referred to stretches of water and was therefore deleted. The transition between the German units “Hauptterrassen – Formationen“ and “Mittelterrassen – Formationen” is marked vaguely in the sheet by DSK 2016. For fid 38 we then decided to set the mean value between these two units as offset. For fid 57 we decided to ignore the overlap between the German “Trias” and Perm in the international stratigraphy and assign the German “Buntsandstein” to the Triassic only.

Finally, information concerning the lithology and stratigraphy had to be reclassified to be used as a base for the stratigraphic and lithological map. For the stratigraphic map, the beginning and offsetting epochs have been summarized in the column “epoch_gen”. For the lithologic map, the lithological information has been categorized in the column “lith_gen” based on the sedimentological process (e.g., fluvial deposits, hillslope deposits, loess) or rock type (e.g., sandstone, dolomite, limestone). Only the vulcanites were classified together regardless of their age. To identify the underlying sedimentological process, we took the regional German units into account. In some cases, the GK 100 and GÜK 300 provided singular labels (e.g., limestone), while in others it combined several lithologies into one label when there are multiple lithologies in one polygon (e.g., limestone, limesandstone, marlstone, siltstone). Therefore, we have formed one category with the singular lithology and another with the remaining lithologies to preserve the maximum information content by also keeping the amount of generalization classes as minimal as possible.

To create the final shapefile, the master data was imported as a vector layer and joined with the attribute table by the column “fid” by using the *join* feature. The result is the shapefile *Ahr_masterdata.*shp that contains all information provided in the master table *Ahr_geology.csv*.

## 6 Creation of an Internationally Valid Chronostratigraphic and Lithologic Map

**Fig. 1:**
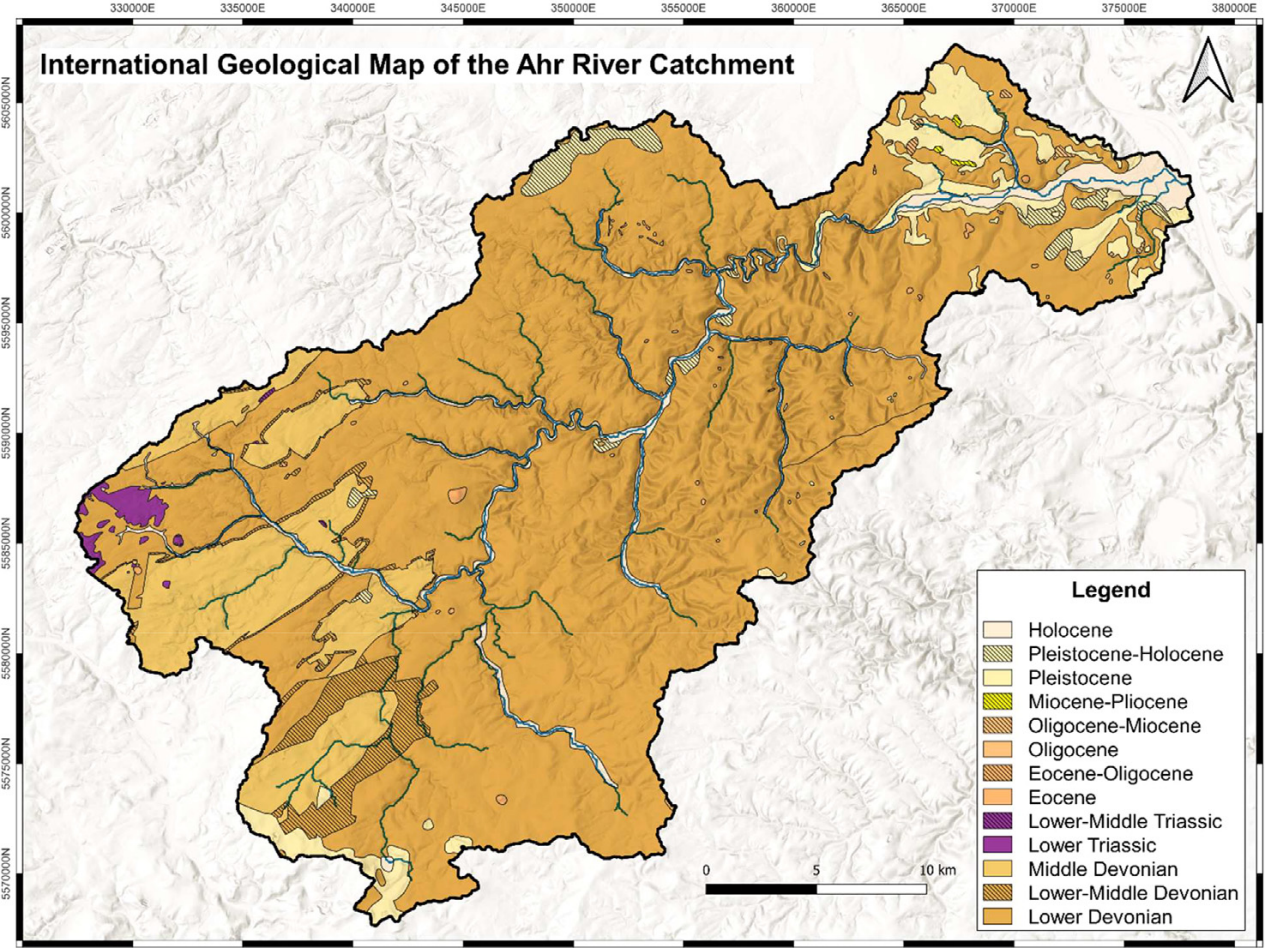
International Geological Map of the Ahr River catchment presenting standardized chronostratigraphic information adapted from the information provided by the federal states of North Rhine-Westphalia [3] and Rhineland-Palatinate [2].

**Fig. 2:**
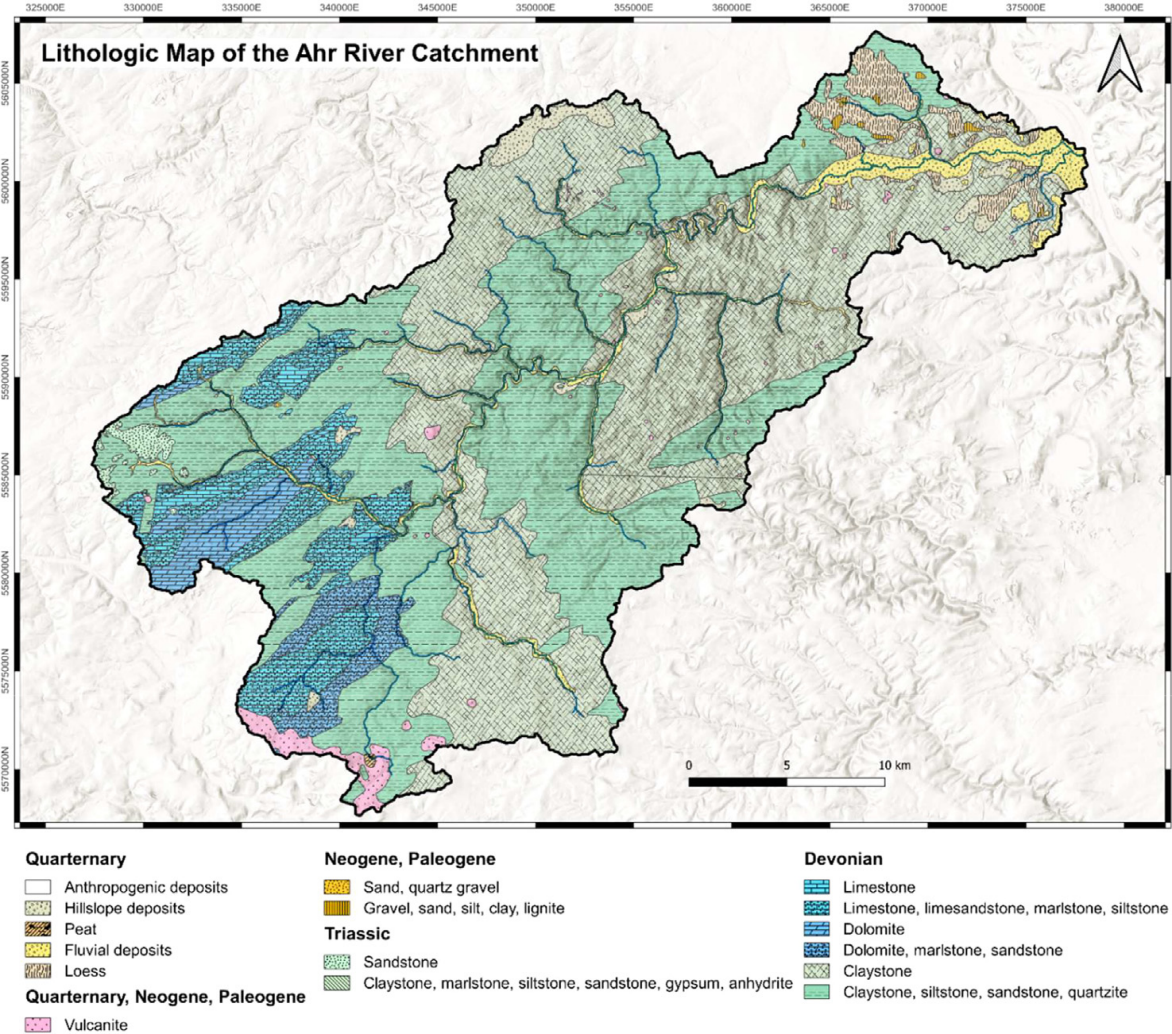
Lithologic Map of the Ahr River catchment adapted from the information provided by the federal states of North Rhine-Westphalia [3] and Rhineland-Palatinate [2].

To create the shapefiles *Ahr_geology.shp* and *Ahr_lithology.shp* as basis for the provided maps, the master data shapefile was once *dissolved* after the column “epoch_gen” and once after the column “lith_gen”. Again, several artifacts had to be removed by using the *Toggle editing Vertex tool* for both new shapefiles. Then the attribute tables were reduced to fewer columns as can be seen in Tables 2 and 3 as the information provided got lost due to dissolving. Furthermore, the stratigraphic order was updated. We then added the symbology for the final outlook of the two maps. For the chronostratigraphic map as seen in Fig. 1 every feature was assigned the color of the epoch according to the *Colour chart* provided by the IUGS [1]. For timespans covering two epochs the color was chosen for the older epoch and a pattern was added to mark the transition. For the lithological map as seen in Fig. 2 every feature was assigned a color as well as a pattern. The colors were assigned according to the *Lith class polygon colors* provided by the United States Geological Survey USGS [6]. The patterns were chosen from the catalogue *Cartographic Standard for Geologic Map Symbolization* provided by the USGS [7] as well.

## Ethics Statements

Not applicable.

## CRediT authorship contribution statement

**Svenja Scholz:** Methodology, Data curation, Writing – original draft. **Henriette Westermann:** Methodology, Data curation, Writing – original draft. **Ronja Hegemann:** Data curation. **Mara Popp:** Data curation. **Tobias Richter:** Data curation. **Markus L. Fischer:** Supervision, Writing – review & editing. **Christoph Zielhofer:** Supervision, Writing – review & editing.

## Data Availability

An international standardized dataset and map of the Ahr River catchment geology, Germany (Original data) (PANGAEA).
